# The efficacy and safety of low-intensity focused ultrasound pulses for prolonged disorders of consciousness: a study protocol for a randomized controlled trial

**DOI:** 10.3389/fneur.2025.1597567

**Published:** 2025-11-06

**Authors:** Yuehong Huang, Qiuyi Yu, Hongxiang Liu, Chenxia Xue, Caroline Schnakers, Steven Laureys, Martin M. Monti, Haibo Di

**Affiliations:** 1 International Vegetative State and Consciousness Science Institute, Hangzhou Normal University, Hangzhou, China; 2 College of Life and Environmental Sciences, Hangzhou Normal University, Hangzhou, China; 3 Basical Department, Medical College, Hangzhou Normal University, Hangzhou, China; 4 Department of Intensive care rehabilitation, Shanghai Yangzhi Rehabilitation Hospital (Shanghai Sunshine Rehabilitation Center), Shanghai, China; 5 Research Institute, Casa Colina Hospital and Centers for Healthcare, Pomona, CA, United States; 6 Coma Science Group, GIGA Consciousness and Centre du Cerveau, University & University Hospital of Liège, Liège, Belgium; 7 Department of Psychology, University of California Los Angeles, Los Angeles, CA, United States; 8 Brain Injury Research Center (BIRC), Department of Neurosurgery, University of California Los Angeles, Los Angeles, CA, United States; 9 Department of Neurosurgery, University of California Los Angeles, Los Angeles, CA, United States

**Keywords:** low-intensity focused ultrasound, prolonged disorders of consciousness, randomized controlled trial, multi-modal measurement, rehabilitation

## Abstract

**Background:**

Recent randomized controlled trials (RCTs) in patients with prolonged disorders of consciousness (pDoC) have yielded limited success. Among them, only studies involving amantadine have provided Class II evidence. The effects of other non-invasive brain stimulation techniques remain inconclusive, largely due to patient heterogeneity and the clinical complexities of implementing such interventions. Low-intensity focused ultrasound pulses (LIFUP), as a novel, non-invasive, and safe neuromodulation technique, have the potential to both stimulate and inhibit deep subcortical structures. This makes LIFUP a promising approach for modulating consciousness and promoting recovery in patients with pDoC. This study aims to evaluate the therapeutic efficacy and safety of LIFUP through a randomized controlled design.

**Methods and analysis:**

Our primary research focus involves conducting multimodal neurofunctional assessments throughout the intervention period. Specifically, we intend to investigate the relationship between Blood Oxygen Level-Dependent (BOLD) signals, electroencephalography (EEG) patterns, thalamic concentrations of glutamate and glutamine (Glx) and gamma-aminobutyric acid (GABA) and behavioral outcomes under two different LIFUP parameter settings (100 Hz transcranial ultrasound stimulation [TUS] and theta-burst TUS [tbTUS]).

**Discussion:**

Through a comprehensive exploration of parameter setting combined with multimodal neurofunctional assessments, this study evaluates both therapeutic potential and safety considerations of ultrasound-based interventions for pDoC. We hypothesize that the two stimulation protocols (100 Hz TUS and tb TUS) will differentially modulate neural connectivity, thalamus activity, and the Glx/GABA balance. The findings may advance evidence-based interventions for pDoC and identify potential neuroplasticity biomarkers to guide future therapeutic strategies.

**Clinical trial registration:**

Chinese Clinical Trial Registry, ChiCTR2400092904. Registered on 26 November 2024.

## 1 Introduction

Consciousness is a fundamental aspect of human existence and has long intrigued both philosophers and scientists alike (1). In a clinical context, Plum and Posner proposed that consciousness can be conceptualized as comprising two principal dimensions: arousal, referring to wakefulness, and awareness, referring to subjective experience or phenomenology (2). However, evaluating the presence of consciousness in individuals who have survived severe brain injury remains a significant challenge. Clinically, the assessment of consciousness and its disorders primarily depends on observable behavior to infer the patient’s level of consciousness (3). Disorders of consciousness (DoC) encompass conditions marked by altered arousal and awareness (4), including coma (a state of complete unresponsiveness without wakefulness) (2), vegetative state (VS) (5), also referred to as Unresponsive Wakefulness Syndrome (UWS) (6) and the minimally conscious state (MCS) (7). The VS is a state of wakefulness without awareness in which there is preserved capacity for spontaneous or stimulus-induced arousal evidenced by sleep–wake cycles and a range of reflexive and spontaneous behaviors (5). In contrast, MCS patients show intermittent signs of awareness and may slowly regain some cognitive function (7). Among patients with prolonged disorders of consciousness (pDoC)(any disorder of consciousness that has continued for at least 4 weeks following sudden onset brain injury) (5), predicting recovery remains fraught with uncertainty. Nevertheless, both in the acute and chronic stages of DoC, there were documented cases of patients gradually regaining consciousness and functional abilities over time^4.5^. This capacity for recovery has drawn significant interest in the neuroscience community (4). Some studies suggested that the nervous system may retain viable neurons even during severe stress states, and that not all neural populations were uniformly affected by the initial insult (8, 9). The body may activate intrinsic protective mechanisms, as observed in patients who regain consciousness after cardiac arrest or COVID-19-related coma (8). A notable study of 50 patients with chronic VS/UWS (duration >6 months) followed for nearly 2 years found that 6 patients (12% of the sample) ultimately recovered consciousness (10). These findings underscored the clinical significance of pursuing effective interventions and treatments aimed at enhancing the potential for recovery in patients with pDoC (11).

Currently, despite a growing landscape of studies investigating non-invasive neuromodulatory treatments for pDoC (12, 13), none has yet been established to systematically improve consciousness levels or functional recovery in this population. First, regarding transcranial direct current stimulation (tDCS) (14), a multicenter randomized clinical trial conducted in 2023 evaluated the effects of tDCS during rehabilitation. While no significant therapeutic effect was observed at the group level, subgroup analysis at a 3-month follow-up revealed notable improvements in patients with minimally conscious state (MCS) and traumatic etiologies. Second, in the field of transcranial magnetic stimulation (TMS) (15), repetitive TMS (rTMS) applied to the dorsolateral prefrontal cortex in 16 patients with DoC (5 MCS and 11 VS/UWS) resulted in increased (the Coma Recovery Scale-Revised) CRS-R scores in 36% of VS/UWS patients and all MCS patients. Moreover, a 2022 study applying 20 Hz rTMS (16) demonstrated significant improvements in consciousness in the real stimulation group compared to the sham group. However, in-depth analysis revealed that only a subset of patients experienced clinically meaningful gains. Third, for transcutaneous auricular vagus nerve stimulation (taVNS), a 2023 randomized double-blind controlled trial (17) involving 57 DoC patients found that while the intervention group showed higher CRS-R scores post-treatment compared to the control group, the difference did not reach statistical significance. Finally, in the realm of Low-intensity focused ultrasound pulses (LIFUP), Monti et al. (18) pioneered the use of non-invasive ultrasound to stimulate the thalamus (100 Hz TUS) in a patient with MCS following traumatic brain injury in 2016. The patient exhibited significant clinical improvement, marking a pivotal moment in the application of LIFUP for DoC. Subsequent studies have reported increases in CRS-R scores in both acute (19) and chronic (20) DoC cases. However, The absence of rigorous comparison with a sham stimulation group limits these findings, highlighting the need for controlled investigations.

LIFUP holds substantial promise as a noninvasive modality for modulating human neural circuits (21). Compared to conventional non-invasive brain stimulation techniques, ultrasound offers unparalleled spatial precision, capable of targeting both cortical and deep subcortical brain structures with a resolution on the order of just a few cubic millimeters. This high degree of focus allows for the precise stimulation of small subcortical regions, such as the thalamus, which previously required invasive deep brain stimulation (DBS) techniques (21). Moreover, LIFUP is regarded as a highly safe method of neuromodulation (22, 23). Given the heterogeneity among patients with DoC, LIFUP presents a promising therapeutic avenue, though its clinical efficacy remains to be confirmed through well-designed randomized controlled trials. The underlying mechanisms of LIFUP are not yet fully elucidated. A prevailing hypothesis suggests that the acoustic pressure delivered by LIFUP induces nanoscale deformation of neuronal membranes, which may modulate the activity of ion channels and mechanosensitive receptors embedded in the membrane (22, 23). In particular, LIFUP may alter the function of mechanosensitive ion channels, influencing the excitability and spontaneous firing rates of neurons, thereby inducing both short-term and long-term neuroplastic changes (21–23). These physiological effects may further result in modulations of cerebral blood flow, the release and uptake of neurotransmitters, and ultimately, alterations in neural circuit function (24–26). As such, LIFUP represents a compelling tool for investigating and potentially restoring consciousness in patients with DoC.

In 2021, Cain et al. (20) conducted a pilot intervention using thalamic LIFUP on three patients with pDoC, of whom two demonstrated significant improvements in behavioral responsiveness compared to baseline. In 2022, a similar intervention (19) was applied to 11 patients with acute DoC, with nine exhibiting behavioral improvements. Both studies reported favorable safety profiles and a high response rate. However, these proof-of-concept case series lacked a sham-controlled condition, limiting the strength of causal inferences. In a separate 2022 study (27), an 80-s train of theta-burst patterned transcranial ultrasound (tbTUS) was applied to the motor cortex of 15 healthy participants. The results revealed elevated corticospinal excitability sustained for up to 30 min, alongside reduced short-interval intracortical inhibition and enhanced intracortical facilitation, suggesting that tbTUS may promote long-term cortical plasticity. Given the operational characteristics of LIFUP, thalamic stimulation using specific parameter sets may likewise elicit excitatory effects and induce plastic changes within thalamic circuits. However, no studies to date have directly compared the effects of different LIFUP parameter configurations, specifically, 100 Hz TUS and tbTUS in patients with pDoC. This represents a critical gap in current knowledge. Investigating whether these parameters produce differential outcomes and how they modulate neural functional connectivity, neurotransmitter dynamics, and EEG signal features is essential. Such insights could optimize treatment protocols and advance our understanding of the neurobiological mechanisms underlying recovery in pDoC.

Based on the above, this study aims to evaluate the efficacy and safety of LIFUP as a potential neuromodulatory treatment. Another major focus of our investigation is to explore the relationship between multimodal neurofunctional assessments and behavioral outcomes in patients with pDoC. We hypothesize that participants receiving LIFUP delivered via either 100 Hz TUS or tbTUS will exhibit significantly improved behavioral recovery metrics compared to sham-controlled participants. Furthermore, we predict that 100 Hz TUS (19, 20) will induce superior neuroplastic effects relative to tbTUS (27), as quantified through multimodal functional connectivity analyses. These differential effects are anticipated to manifest as: (1) increased phase-locking value (PLV) in whole-scalp or region-specific high-density electroencephalography (HD-EEG), and (2) enhanced effective connectivity (EC) in task-based functional magnetic resonance imaging (fMRI), particularly within targeted neuromodulation networks.

## 2 Methods and analysis

### 2.1 Study design

This is a single-site, controlled randomized trial conducted at the Intensive Care Rehabilitation Ward of Shanghai Yangzhi Rehabilitation Hospital, also known as the Shanghai Sunshine Rehabilitation Center. In this parallel-group study, 78 patients with pDoC will be prospectively randomized in a 1:1:1 ratio to one of three arms: Group A (100 Hz TUS), Group B (tbTUS), or Group C (sham LIFUP stimulation).

All enrolled patients will receive two sessions of TUS treatment whether it is real or sham stimulation. In addition to CRS-R assessments, comprehensive neurophysiological and neuroimaging assessments, including HD-EEG, Somatosensory Evoked Potentials (SSEPs), fMRI, proton magnetic resonance spectroscopy (MRS) and positron emission tomography-computed tomography (PET-CT), will be performed at baseline (pre-treatment) and follow-up (post-treatment) time points. Longitudinal outcome measures will be assessed at three timepoints: 1 month (30 ± 7 days), 3 months (90 ± 14 days), and 6 months (180 ± 30 days) following the final intervention.

**Figure 1:**
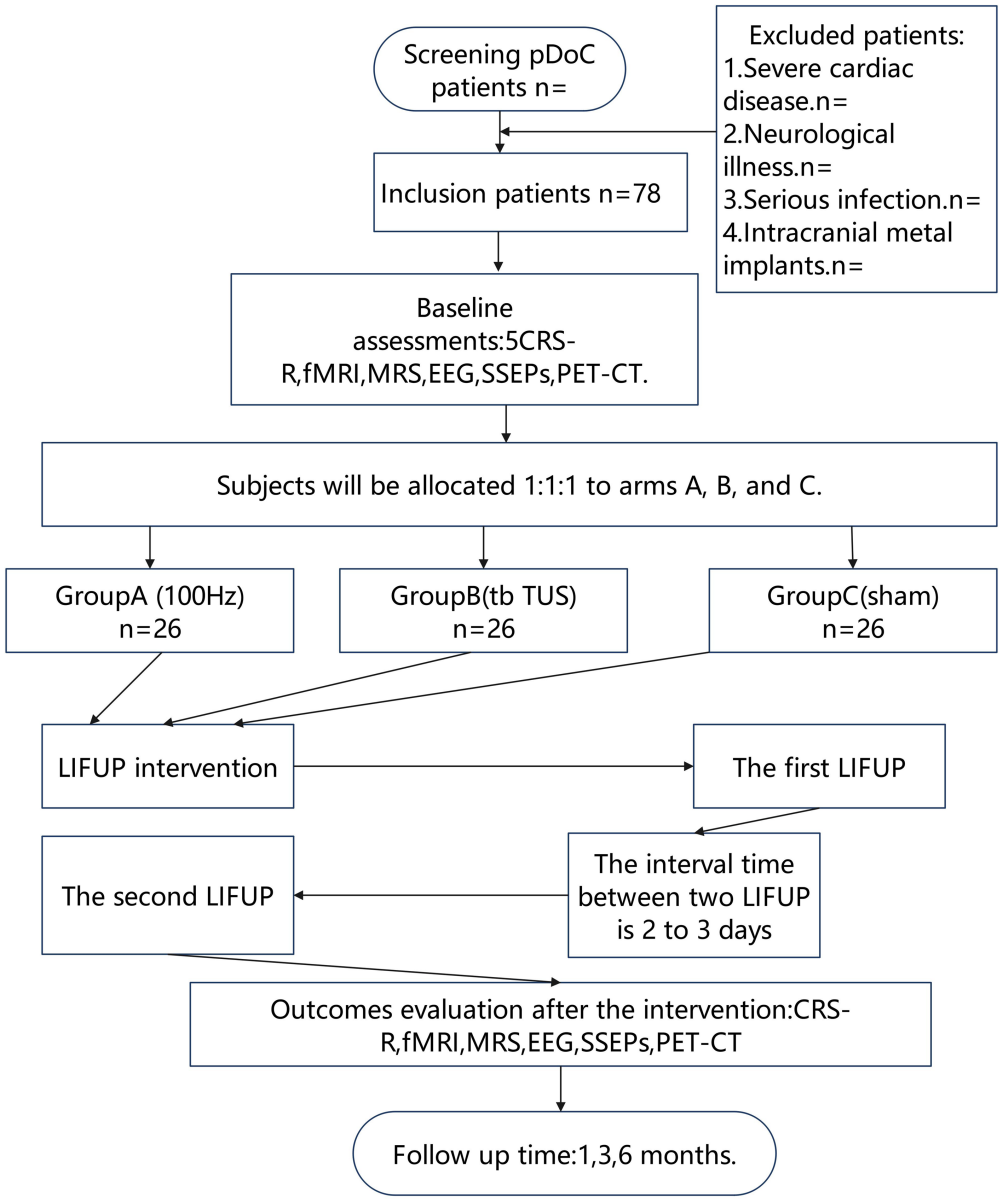
Flow diagram of the study.

During each treatment session, vital signs including body temperature, heart rate, blood pressure, and respiratory rate will be monitored to ensure safety. The vital signs of the patients will be continuously monitored 24 h after the intervention. The trial will be supervised by a team comprising at least one neurologist, one neurorehabilitation specialist, and associated research staff. All procedures and evaluations will adhere strictly to the study protocol. A schematic flow chart of the trial design is provided in Figure 1.

### 2.2 Selection of subjects

#### 2.2.1 Inclusion criteria

1. Participants must have sustained their injury or onset more than 28 days prior to enrollment;
2. Participants must be over 18 years of age;
3. Participants must exhibit a behavioral profile consistent with either a VS/UWS or a MCS, as assessed by the CRS -R;
4. Participants must not have experienced any significant fluctuations in their medical condition during the past week. Common causes of instability include fever exceeding 37.3 °C within the past 7 days, epileptic seizures, paroxysmal sympathetic hyperactivity, and inability to maintain adequate oxygenation for 40 min. The above conditions will be assessed by two ICU physicians based on the patient’s status over the recent week.

#### 2.2.2 Exclusion criteria

1. Participants with significant/severe cardiac disease;
2. Participants with a history of neurological illness prior to the current injury;
3. Participants who are unable to safely undergo MRI scanning due to a severe infection;
4. Participants with intracranial metal implants.

All patients will be recruited from the intensive care and rehabilitation wards. The study will be conducted from March 2025 to December 2025. Potential participants will be identified by reviewing electronic medical records within the hospital information system (HIS). Researchers will explain the details of the study to the patients’ legal guardians. Upon obtaining consent, a neurologist will evaluate each candidate based on the inclusion and exclusion criteria. Those who meet the eligibility requirements will be formally invited to participate in the study and will undergo a comprehensive neurological examination, followed by a rehabilitation assessment, prior to the intervention.

#### 2.2.3 Sample size

For the behavioral assessment using the CRS-R, this study adopts a randomized controlled intervention design. Repeated measures will be analyzed using ANOVA to account for potential confounding variables. Based on a power analysis conducted using G*Power, with an effect size of 0.40, a significance level (*α*) of 0.05, statistical power (1 − *β*) of 0.80, a two-tailed test (α = 0.05), and an equal allocation ratio among the three groups (N1: N2: N3 = 1:1:1), the required each group sample size is calculated to be 18. After applying Bonferroni correction, the significance threshold for individual comparisons should be adjusted to α = 0.0167. To maintain this corrected alpha level, each group would require a sample size of 22. Considering a potential 15% dropout or exclusion rate, a minimum of 76 participants is required. Therefore, the study will enroll a total of 78 participants: 26 in Group A, 26 in Group B, and 26 in Group C. In the event of patient withdrawal or death during the study, we will implement standardized procedures. All withdrawal cases will be documented systematically, including: (1) voluntary withdrawal (participant-initiated consent revocation), (2) loss to follow-up (≥3 consecutive contact attempts failed), (3) clinical deterioration (≥2-point GCS decrease sustained for 48 h), and (4) investigator-determined withdrawal (with protocol-specified justification). For data management, all collected pre-withdrawal data will be retained per intention-to-treat principles, with final evaluable assessments carried forward for primary analysis. Mortality cases will trigger immediate reporting to the Ethics Committee of Shanghai Yangzhi Rehabilitation Hospital (Shanghai Sunshine Rehabilitation Center), followed by survival analysis using Kaplan–Meier methodology and independent adjudication of causes by a clinical endpoint committee. Statistical approaches will include sensitivity analyses (completers vs. non-completers) and multiple imputation for missing data.

#### 2.2.4 Randomization

Randomization will be performed using the random number generation function in the R programming language (set.seed). All eligible participants will be randomly assigned to one of three groups in a 1:1:1 ratio: Group A (100 Hz TUS), Group B (tbTUS), and Group C (sham stimulation). To ensure allocation concealment and fairness, a randomization sequence will be generated by an independent researcher who is not involved in the trial’s implementation or outcome evaluation. The randomization sequence will be kept confidential and will not be disclosed to outcome assessors or statisticians. Baseline assessments will be completed after group assignment. Healthcare providers and therapists involved in the participants’ care, including acupuncturists and cognitive therapists, will remain blinded to group allocation to minimize bias.

### 2.3 Interventional methods

**Figure 2:**
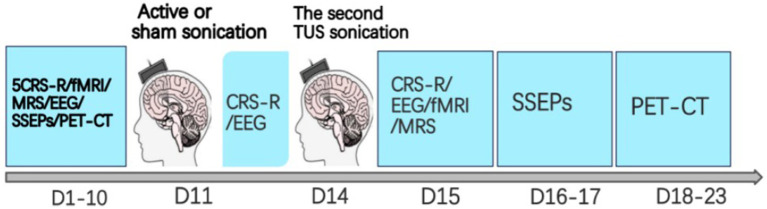
The LIFUP intervention protocol.

After initial screening and evaluations, all patients in the three groups will undergo two sessions of LIFUP. Prior to LIFUP, K-Plan simulation will be performed to determine the optimal stimulation trajectory. Prior to the intervention, baseline assessments will be conducted within a 7- to 10-day window, including five repeated CRS-R evaluations, HD-EEG, SSEPs, fMRI, MRS and PET-CT. The first session of LIFUP will be administered between days 11 and 13, with the second session delivered after a 2- to 3-day interval. Each stimulation session will be conducted according to its corresponding parameter specific duration. CRS-R evaluations will be conducted 1 h before and after each session of LIFUP. Following the second session of LIFUP, repeat assessments including EEG, SSEPs, fMRI, MRS, and PET-CT will be conducted, with all evaluations completed within 5–7 days post-intervention. The intervention phase will last approximately 23 days in total. The entire process of examination and treatment is shown in Figure 2.

#### 2.3.1 Parameters

**Figure 3:**
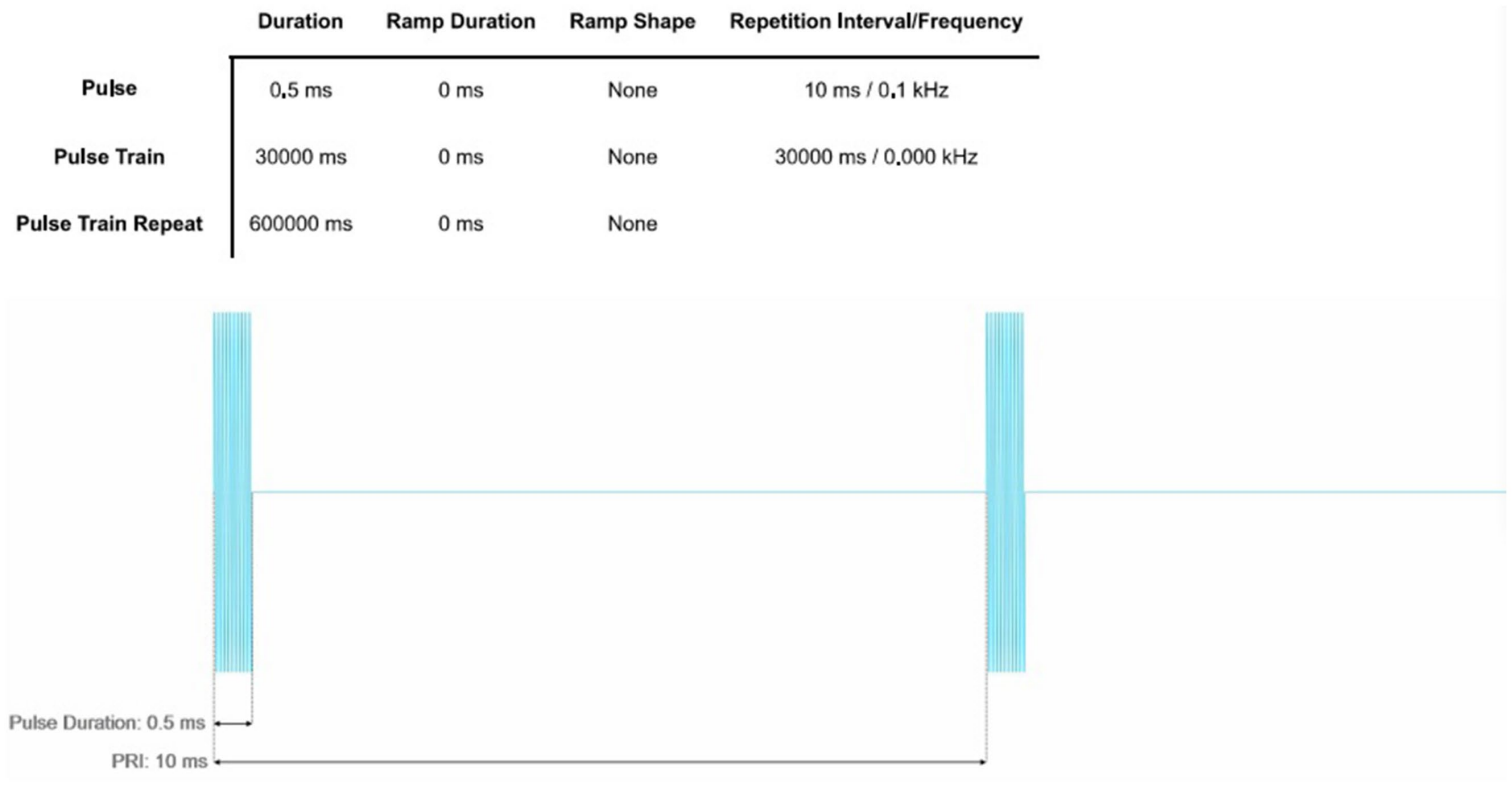
Pulse timing parameters of 100 Hz TUS.

For the stimulation protocol in Group A (100 Hz TUS), LIFUP will be applied with the following parameters: Fundamental Frequency (FF):500 kHz, Pulse Repetition Frequency (PRF): 100 Hz, Pulse Width(PW):0.5 ms, Duty Cycle (DC):5%, Sonication Duration (SD):30 s, Number of Sonication:20, Spatial-peak Pulse-Average Intensity (ISPPA):14.39 W/cm (2), and Spatial-peak Temporal-Average Intensity (ISPTA):719.73 mW/cm (2). Peak rarefactional pressure:0.68 MPa. The pulse timing parameters of 100 Hz TUS are shown in Figure 3.

**Figure 4:**
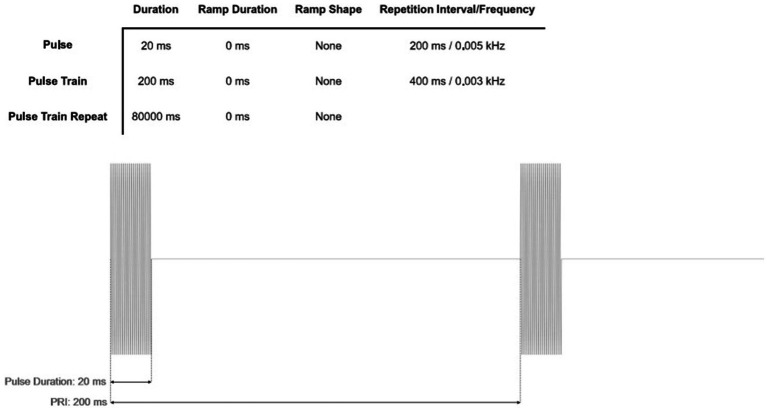
Pulse timing parameters of tbTUS.

Under stimulation condition Group B, referred to as tb TUS following protocol (27), the parameters will be set as follows: FF of 500 kHz, PRF of 5 Hz, PW of 20 ms, DC of 10%, SD of 200 ms, with a total of 400 sonication. ISPPA is 7.19 W/cm (2), and the ISPTA is 719.73 mW/cm (2). Peak rarefactional pressure:0.48 MPa. The pulse timing parameters of tbTUS are shown in Figure 4. Group C will undergo the tbTUS protocol, but the transducer’s power supply will remain deactivated. In order to minimize possible sound-related confounds during TUS (28), white noise will be delivered to participants through earbuds while sonication is administered. The neural activity and safety of both stimulation protocols (100 Hz TUS and tbTUS) have been validated in several previous studies (19, 20, 27, 29). The energy levels employed in this study are well below the limits set by the U. S. Food and Drug Administration (FDA) for diagnostic ultrasound interventions through the human skull (mechanical index, MI ≤ 1.9; spatial-peak pulse-average intensity, ISPPA≤190 W/cm (2)) (30).

#### 2.3.2 LIFU instrument

The LIFU signals were produced by a four-element ring array transducer (NeuroFUS Pro, DAPX-500, Brainbox Ltd., Cardiff, UK) featuring a 64 mm outer aperture diameter and radius of curvature. This transducer was connected to a programmable RF amplifier (Transducer Power Output System, TPO-203, Brainbox Ltd., Cardiff, UK) and driven at 500 kHz by a 4-channel power amplifier (Sonic Concepts). According to the manufacturer’s test results, the output power and phase of each element were precisely controlled to generate an in-water focal pressure of 948.7 kPa (equivalent to a pulse-average spatial peak intensity, ISPPA, of 30 W/cm (2)) at a focal distance of 53.6 mm. The measured -3 dB focal size in water was 19.5 mm (lateral) by 53.0 mm (axial). If the average skull attenuation (insertion loss) is −10 dB (31), the estimated intracranial acoustic parameters include a peak focal pressure of 300 kPa and an ISPPA of 15 W/cm (2). The actual insertion loss varies depending on individual patient skull characteristics. For precise targeting, transducer positioning was guided by neuronavigation (Brainsight, Rogue Research Inc.), with real-time tracking accounting for individual skull attenuation characteristics that inherently reduce the effective focal intensity.

#### 2.3.3 Target

Based on foundational research involving DBS in non-human primates (32), clinical DBS case studies (33), mechanistic insights (34), and accumulated therapeutic experience (19, 20), the stimulation targets for this study are selected from the medial and intralaminar thalamic nuclei group of the ALL3 atlas (35). Specifically, these include the Reuniens nucleus (Thal_RE), the medial magnocellular portion of the mediodorsal nucleus (Thal_MDm), and the lateral portion of the mediodorsal nucleus (Thal_MDl), as well as the intralaminar thalamic nuclei (Thal_IL). The stimulation will target either the left or right side of the thalamus, in accordance with the anatomical references outlined in the ALL3 atlas (35).

**Figure 5:**
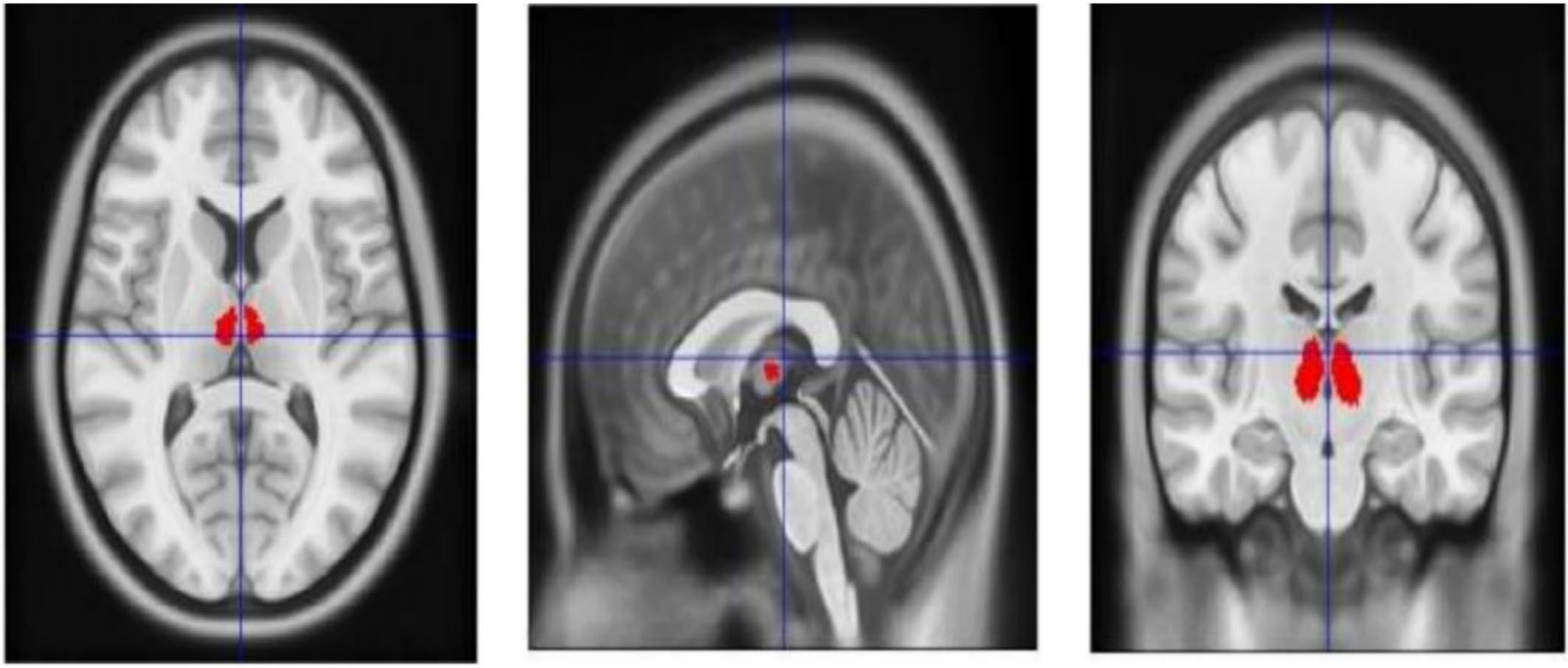
The target of this study is delineated on the MNI template, with the red area specifically indicating the region of interest.

The target acquisition process involves the following steps:

1. Data conversion: Digital Imaging and Communications in Medicine (DICOM) data are first converted into the standardized Neuroimaging Informatics Technology Initiative (NIfTI) format.
2. Segmentation: The segmentation function in SPM12 (36) is applied to each three dimensional (3D) T1-weighted MRI image to segment gray matter, white matter, and cerebrospinal fluid.
3. Thalamic mask extraction and normalization: A thalamic mask is extracted from the ALL3 template and then normalized to the individual’s T1-weighted MRI for precise anatomical localization (demonstrative images are provided in Figure 5).

In cases where patients exhibit skull defects, LIFUP intervention may be considered on the intact side of the skull. However, the absence of skull can lead to a significant increase up to 50% in ultrasound energy at the target site. This amplification compromises the standardization, stability, and safety of the intervention. Therefore, LIFUP is not recommended for patients with large skull defects or those who have undergone cranioplasty involving metallic implants. Additionally, caution should be exercised when considering LIFUP for patients with implanted ventriculoperitoneal (VP) shunts for hydrocephalus.

#### 2.3.4 Simulations (k-Plan)

**Figure 6:**
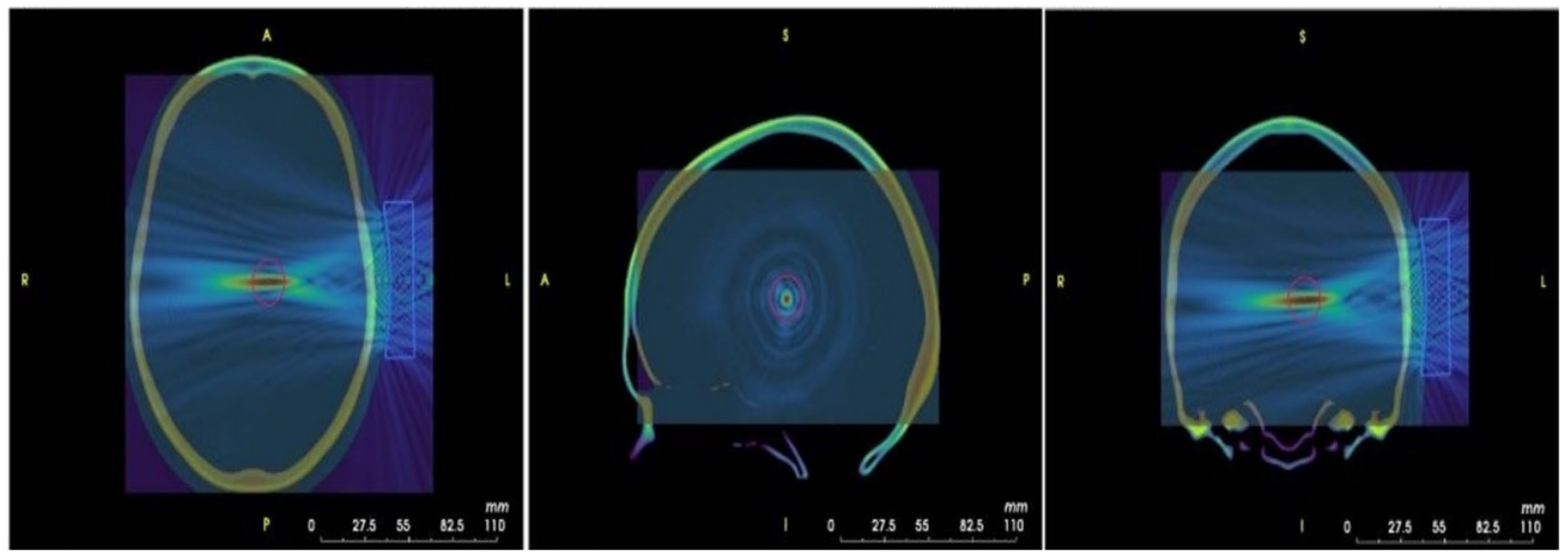
Pressure maps derived from ultrasound modeling superimposed on the MRI image of the MNI template, which was obtained from k-Plan.

We will utilize k-Plan software to conduct our simulations, using a transducer model configured according to the physical properties of the NeuroFUS transducer and the phase settings specified for the TPO unit. Given the deep subcortical location of the left and right thalamus, we will simulate the ultrasound beam with a focal depth tailored to the target distance determined by Brainsight, which individually reflects the focal depth required for each participant’s anatomical structure. Transcranial simulations targeting the left or right thalamus will be performed for each participant. All patients will have undergone a cranial CT scan during their hospital stay, which will be used for default CT calibration. We will use 3D Slicer to co-register the head CT data with the participant’s T1-weighted MRI to ensure precise anatomical alignment. Following the simulation, relevant reports and results will be generated. Based on these outcomes, we will identify the optimal stimulus location. Subsequently, Brainsight software will be used to guide and implement the stimulation procedure in accordance with the simulation results. (Illustrative images are provided in Figure 6). The simulated images displayed in Figure 6 were generated using standardized neuroimaging templates. The MR template employed in k-Plan corresponds to the ICBM 1522009c Nonlinear Symmetric template (1 × 1 × 1 mm resolution; filename: mni_icbm152_t1_tal_nlin_sym_09c.nii), as described by Fonov et al. (37). The CT template implementation follows the methodology outlined by Rorden et al. (38). For additional technical details regarding these planning images, please refer to the official k-Plan documentation available at.^1^

#### 2.3.5 Procedure

Ultrasound transmission gel (Hainuohai, Qingdao Hainuo Bioengineering Co., Ltd., China) will be applied to each participant’s scalp to ensure optimal acoustic coupling. A gel pad (Jiangkangtang, Zhengzhou Kangyijian Medical Devices Co., Ltd., China) will be used in conjunction with the transducer probe. Care will be taken to eliminate any air bubbles between the transducer surface and the participant’s head, as these may interfere with ultrasound transmission. Neuro-navigation will be performed using Brainsight software (version 2.5.3, Rogue Research Inc., Montreal, Quebec, Canada) based on each participant’s anatomical T1-weighted MRI scan. The target lesion depth will be adjusted for each individual according to the simulation scheme generated in K-Plan. During the LIFUP sessions, the navigation software will be used to continuously identify and monitor the targeted stimulation regions. The software will also be used to locate sensor coordinates and detect any deviations from the predetermined focal point.

#### 2.3.6 Safety assessments and follow-up

We will make sure to get approval from key family members before proceeding. In terms of safety, we will record important parameters such as heart rate, blood oxygen and blood pressure during LIFU intervention and imageological examination data collection. In addition, any adverse events that occurred to the patients during the course of the study will be recorded. Patients will undergo follow-up assessments using CRS-R at 1, 3, and 6 months after LIFUP intervention treatment, and if possible, re-examination of fMRI and EEG will be conducted.

### 2.4 Outcome measures

The primary outcome of this study is the recovery of consciousness measured by CRS-R and CRS-R_index_. Secondary outcomes will include the assessment of neurophysiological changes through multimodal neuroimaging and electrophysiological measures (fMRI, MRS, EEG) to evaluate intervention efficacy and safety. Additionally, SSEPs and PCT-CT measurements will be obtained at both baseline and endpoint assessments. These measurements will provide valuable insights into the impact of the study and aid in further understanding the research findings. Any adverse outcomes or side effects will be documented throughout the treatment process.

#### 2.4.1 Primary outcome detection (behavioral data evaluation)

CRS-R and CRS-R_Index_: at the individual level, the CRS-R will be used to evaluate the progress of patients with pDoC. The CRS-R comprises 23 items across six subscales that assess auditory, visual, motor, oromotor/verbal, communication, and arousal functions. Changes in patient status will be assessed by comparing pre- and post-treatment scores for each subscale. At the group level, the CRS-R_Index_ score will be used to evaluate behavioral reactivity across both intervention and control groups, as it is considered to provide a more representative measure of functional recovery. CRS-R assessments will be conducted at baseline, before the intervention, and after each treatment session to capture the best observed performance for each patient. For patients who undergo two sessions of LIFUP, scores will be averaged and included in the group-level analysis.

#### 2.4.2 Secondary outcome detection (image and electrophysiological data assessment)

At baseline and following treatment of LIFUP, patients will undergo a series of MRI scans using a 3.0 T Siemens VIDA scanner (Siemens Healthineers, Erlangen, Germany) equipped with a 64-channel head coil. The experiment will acquire the following sequence of scans: T1-weighted imaging, BOLD fMRI, DTI, and 1H-MRS (see Supplementary material 1 for details). Previous psychophysiological interaction (PPI) analysis19 revealed a more complex change in connectivity between the thalamus and the rest of the brain during LIFU application. The primary objective of this study is to investigate potential changes in thalamocortical connectivity, particularly between the thalamus and prefrontal/parietal cortices, before and after intervention. Glx/GABA concentrations will be quantified using 1H-MRS. While prior studies24,25 have reported stimulation-induced Glx or GABA level changes in cortical regions, these findings remain largely restricted to superficial cortical areas. In this study, we aim to examine whether different stimulation parameters induce measurable changes in concentrations of Glx/GABA within the medial and intralaminar nuclei of the thalamus. This may provide further insight into the underlying neural mechanisms of recovery in patients with DoC.

Additionally, participants will undergo a head PET-CT scan using a Vereos PET-CT scanner (Philips, Netherlands). Each subject will receive an intravenous injection of fluorodeoxyglucose (FDG), dosed at 0.1 mCi/kg based on body weight. After injection, participants will rest quietly in a dimly lit room with eyes closed for 60 min before the scan. Regional changes in glucose metabolism may serve as direct indicators of treatment efficacy and help predict clinical outcomes. Similar metabolic investigations have been reported in studies focusing on the hippocampus (39) (more detailed information regarding fMRI and PET-CT procedures can be found in the Supplementary material 1).

SSEPs will be assessed during the baseline phase prior to stimulation of LIFUP. A follow-up SSEPs evaluation will be conducted 1 day post-treatment during the experimental phase. Drawing on previous protocols involving thalamic TUS stimulation in healthy individuals (40), SSEPs are proposed as a useful indicator for monitoring treatment efficacy and prognostic outcomes.

Both resting-state and task-related EEG recordings will be obtained once on the day before and once on the day after intervention of LIFUP. EEG serves as a critical diagnostic and prognostic tool in patients with pDoC. To date, no studies have systematically reported changes in EEG functional connectivity, complexity measures, or spectral power before and after treatment of LIFUP in this population. Additionally, task-evoked EEG responses following intervention of LIFUP have not yet been explored. This study aims to bridge this gap by investigating EEG-based changes in neural dynamics and connectivity associated with LIFUP, thereby offering new insights into its therapeutic mechanisms in pDoC (more detailed information on SSEPs and EEG protocols is provided in the Supplementary material 1).

### 2.5 Data analysis

We will perform a comprehensive analysis combining behavioral outcomes of CRS-R_index_ (R code available at)^2^ and neuroimaging data (fMRI, MRS, PET-CT). Statistical analyses will be conducted at both the individual and group levels, comparing outcomes before and after treatment, as well as between intervention and control groups. The main statistical approach will include repeated-measures ANOVA to examine within- and between-group effects, accounting for potential confounding variables. Continuous variables will be analyzed using paired or independent sample t-tests, while categorical variables will be assessed using the Mann–Whitney U test. Chi-square tests or Fisher’s exact tests will be employed to evaluate and document adverse events. To explore associations between neurotransmitter concentrations and other outcomes, regression analyses will be conducted. For SSEPs, changes in amplitude and latency before and after treatment will be compared using t-tests. EEG data will be analyzed using EEGLAB (41) to evaluate both local and cross-regional functional connectivity metrics, including phase locking value (PLV) and power spectral density (PSD). Functional MRI assessments will be conducted using SPM12 (36) to examine seed-to-seed functional and effective connectivity during both resting-state and task-based conditions. MRS will be primarily employed to compare pre- and post-treatment changes in Glx and GABA concentrations within the stimulated brain regions. Additionally, PET-CT imaging will be used to assess alterations in glucose metabolism, both in the stimulated areas and across the whole brain, following the intervention. For fMRI or EEG data analysis, we will implement FDR or FWER correction for multiple comparisons, but will not utilize machine learning validation approaches.

## 3 Discussion

Based on the results of our study (Supplementary material 4), the patient showed improved behavioral scores, though not as significantly as those observed in patients with DoC who underwent surgery of DBS (33). Given that our data is limited to a single case, further studies are needed to assess both the efficacy and safety of this approach (LIFUP). Currently, no studies provide Class I evidence regarding noninvasive neuromodulation in patients with pDoC. With the advancement of stereotactic neurosurgery in treating neurological disorders (42), DBS emerged in the 20th century through pioneering case reports involving patients with DoC (43). Most interventions of DBS have targeted patients in a state of VS/UWS, primarily focusing on the thalamus. However, outcomes had generally been underwhelming, and more importantly, this technique was not widely applicable to patients with pDoC (44). More recently, in 2023, Schiff et al. applied DBS therapy to five patients with severe traumatic brain injury (msTBI), leading to enhanced executive functioning (45). Building on these studies, LIFUP presents a non-invasive technique capable of penetrating the skull and targeting subcortical structures. This makes it a promising alternative for patients who are not suitable candidates for DBS, and it may also serve as a preoperative assessment tool for the suitability of DBS. However, due to the still unclear mechanisms underlying the onset and progression of DoC, the use of LIFUP as a standalone treatment in pDoC remains at an exploratory stage.

The mechanism underlying recovery from pDoC remains poorly understood. At the level of neural functional connectivity, pDoC was associated with severe disruptions in resting-state network connectivity, particularly within higher-order networks (46). Interestingly, has been observed in the limbic system including the orbitofrontal cortex, insula, hypothalamus, and ventral tegmental area in patients with VS/UWS (47). Such hyperconnectivity may reflect early-stage injury responses, as brain injury often disrupted normal neural connectivity and may initially provoked compensatory overactivation (48). As described in studies of DBS, stimulation of the central thalamus could both increase and decrease functional connectivity, suggesting that the brain may adjust connectivity toward a balanced, homeostatic state during recovery (49). While tbTUS demonstrate excitatory (27) cortical effects, its deep nuclear actions appear more complex. Notably, recordings of local field potentials in the internal globus pallidus during intervention of 120 s tbTUS in DBS-implanted patients revealed enhanced theta and beta activity, though the underlying excitatory/inhibitory mechanisms remain to be elucidated (50). This temporal and spatial complexity suggests ultrasound’s therapeutic effects may vary by both disease stage and anatomical target. Finally, we suggest that the mechanisms underlying the recovery of DoC require more precise experimental designs of TUS. In the future, this approach could potentially be combined with DBS-implanted patients.

To our knowledge, this is the first randomized controlled trial to investigate the effects and safety of LIFUP on patients with pDoC. We aim to compare the effects of two different parameter sets (100 Hz TUS and tbTUS), and to explore the relationship between behavioral recovery in pDoC and BOLD and EEG signal changes, as well as thalamic concentrations of Glx/GABA. These findings may contribute to a better understanding of the neurobiological mechanisms underlying recovery in pDoC.

However, it should be noted that this is a single-center clinical trial. Future multicenter studies with larger sample sizes will be necessary to validate these results. Moreover, whether the optimal stimulation target should be the left thalamus or bilateral thalami remains an open question, requiring further clinical investigation.

## Footnotes

^1^ https://dispatch.k-plan.io/static/docs/planning-images.html

^2^ https://github.com/Annen/CRS-R/blob/master/CRS-R_index.R

## References

[1] Bernat JL. Chronic disorders of consciousness. Lancet. (2006) 367:1181–92. doi: 10.1016/S0140-6736(06)68508-5, 16616561

[2] Posner JB Saper CB Schiff ND Plum F. Plum and Posner’s diagnosis of stupor and coma. New York: Oxford University Press (2007).

[3] Arciniegas DB Gurin LJ Zhang B. Structural and functional neuroanatomy of Core consciousness: a primer for disorders of consciousness clinicians. Phys Med Rehabil Clin N Am. (2024) 35:35–50. doi: 10.1016/j.pmr.2023.09.002, 37993192

[4] Edlow BL Claassen J Schiff ND Greer DM. Recovery from disorders of consciousness: mechanisms, prognosis and emerging therapies. Nat Rev Neurol. (2021) 17:135–56. doi: 10.1038/s41582-020-00428-x, 33318675 PMC7734616

[5] Royal College of Physicians. Prolonged disorders of consciousness following sudden onset brain injury. National clinical guidelines. London: Royal College of Physicians (2020).

[6] Laureys S Celesia GG Cohadon F Lavrijsen J León-Carrión J Sannita WG . European task force on disorders of consciousness. Unresponsive wakefulness syndrome: a new name for the vegetative state or apallic syndrome. BMC Med. (2010) 8:68. doi: 10.1186/1741-7015-8-68, 21040571 PMC2987895

[7] Giacino JT Ashwal S Childs N Cranford R Jennett B Katz DI . The minimally conscious state: definition and diagnostic criteria. Neurology. (2002) 58:349–53. doi: 10.1212/WNL.58.3.349, 11839831

[8] Schiff ND Brown EN. Protective down-regulated states in the human brain: a possible lesson from COVID-19. Proc Natl Acad Sci USA. (2022) 119:e2120221119. doi: 10.1073/pnas.2120221119, 36343241 PMC9674211

[9] Forgacs PB Devinsky O Schiff ND. Independent functional outcomes after prolonged coma following cardiac arrest: a mechanistic hypothesis. Ann Neurol. (2020) 87:618–32. doi: 10.1002/ana.25690, 31994749 PMC7393600

[10] Estraneo A Moretta P Loreto V Lanzillo B Santoro L Trojano L. Late recovery after traumatic, anoxic, or hemorrhagic long-lasting vegetative state. Neurology. (2010) 75:239–45. doi: 10.1212/WNL.0b013e3181e8e8cc, 20554941

[11] Schiff ND. Toward an interventional science of recovery after coma. Neuron. (2024) 112:1595–610. doi: 10.1016/j.neuron.2024.04.027, 38754372 PMC11827330

[12] Schnakers C Monti MM. Disorders of consciousness after severe brain injury: therapeutic options. Curr Opin Neurol. (2017) 30:573–9. doi: 10.1097/WCO.0000000000000495, 28901969

[13] Thibaut A Schiff N Giacino J Laureys S Gosseries O. Therapeutic interventions in patients with prolonged disorders of consciousness. Lancet Neurol. (2019) 18:600–14. doi: 10.1016/S1474-4422(19)30031-6, 31003899

[14] Thibaut A Fregni F Estraneo A Fiorenza S Noe E Llorens R . Sham-controlled randomized multicentre trial of transcranial direct current stimulation for prolonged disorders of consciousness. Eur J Neurol. (2023) 30:3016–31. doi: 10.1111/ene.15974, 37515394

[15] Xia X Bai Y Zhou Y Yang Y Xu R Gao X . Effects of 10 Hz repetitive transcranial magnetic stimulation of the left dorsolateral prefrontal cortex in disorders of consciousness. Front Neurol. (2017) 8:182. doi: 10.3389/fneur.2017.00182, 28515709 PMC5413493

[16] Fan J Zhong Y Wang H Aierken N He R. Repetitive transcranial magnetic stimulation improves consciousness in some patients with disorders of consciousness. Clin Rehabil. (2022) 36:916–25. doi: 10.1177/02692155221089455, 35322709

[17] Zhou YF Kang JW Xiong Q Feng Z Dong XY. Transauricular vagus nerve stimulation for patients with disorders of consciousness: a randomized controlled clinical trial. Front Neurol. (2023) 14:1133893. doi: 10.3389/fneur.2023.1133893, 36937511 PMC10017768

[18] Monti MM Schnakers C Korb AS Bystritsky A Vespa PM. Non-invasive ultrasonic thalamic stimulation in disorders of consciousness after severe brain injury: a first-in-man report. Brain Stimul. (2016) 9:940–1. doi: 10.1016/j.brs.2016.07.008, 27567470

[19] Cain JA Spivak NM Coetzee JP Crone JS Johnson MA Lutkenhoff ES . Ultrasonic deep brain neuromodulation in acute disorders of consciousness: a proof-of-concept. Brain Sci. (2022) 12:428. doi: 10.3390/brainsci12040428, 35447960 PMC9032970

[20] Cain JA Spivak NM Coetzee JP Crone JS Johnson MA Lutkenhoff ES . Ultrasonic thalamic stimulation in chronic disorders of consciousness. Brain Stimul. (2021) 14:301–3. doi: 10.1016/j.brs.2021.01.008, 33465497

[21] Arulpragasam AR van Wout-Frank M Barredo J Faucher CR Greenberg BD Philip NS. Low intensity focused ultrasound for non-invasive and reversible deep brain neuromodulation-a paradigm shift in psychiatric research. Front Psychiatry. (2022) 13:825802. doi: 10.3389/fpsyt.2022.825802, 35280168 PMC8907584

[22] Tyler WJ. The mechanobiology of brain function. Nat Rev Neurosci. (2012) 13:867–78. doi: 10.1038/nrn3383, 23165263

[23] Baek H Pahk KJ Kim H. A review of low-intensity focused ultrasound for neuromodulation. Biomed Eng Lett. (2017) 7:135–42. doi: 10.1007/s13534-016-0007-y, 30603160 PMC6208465

[24] Yaakub SN White TA Roberts J Martin E Verhagen L Stagg CJ . Transcranial focused ultrasound-mediated neurochemical and functional connectivity changes in deep cortical regions in humans. Nat Commun. (2023) 14:5318. doi: 10.1038/s41467-023-40998-0, 37658076 PMC10474159

[25] Zhang T Guo B Zuo Z Long X Hu S Li S . Excitatory-inhibitory modulation of transcranial focus ultrasound stimulation on human motor cortex. CNS Neurosci Ther. (2023) 29:3829–41. doi: 10.1111/cns.14303, 37309308 PMC10651987

[26] Rodriguez-Rojas R Machado C Alvarez L Carballo M Estevez M Perez-Nellar J . Zolpidem induces paradoxical metabolic and vascular changes in a patient with PVS. Brain Inj. (2013) 27:1320–9. doi: 10.3109/02699052.2013.794961, 23924270

[27] Zeng K Darmani G Fomenko A Xia X Tran S Nankoo JF . Induction of human motor cortex plasticity by Theta burst transcranial ultrasound stimulation. Ann Neurol. (2022) 91:238–52. doi: 10.1002/ana.26294, 34964172

[28] Kop BR Shamli Oghli Y Grippe TC Nandi T Lefkes J Meijer SW . Auditory confounds can drive online effects of transcranial ultrasonic stimulation in humans. eLife. (2024) 12:RP88762. doi: 10.7554/eLife.88762, 39190585 PMC11349300

[29] Yoo SS Kim H Min BK Franck E Park S. Transcranial focused ultrasound to the thalamus alters anesthesia time in rats. Neuroreport. (2011) 22:783–7. doi: 10.1097/WNR.0b013e32834b2957, 21876461 PMC3174273

[30] Duck FA. Medical and non-medical protection standards for ultrasound and infrasound. Prog Biophys Mol Biol. (2007) 93:176–91. doi: 10.1016/j.pbiomolbio.2006.07.008, 16965806

[31] Gimeno LA Martin E Wright O Treeby BE. Experimental assessment of skull aberration and transmission loss at 270 kHz for focused ultrasound stimulation of the primary visual cortex. IEEE Int Ultrason Symp, IEEE. (2019) 2019:556–9. doi: 10.1109/ULTSYM.2019.8926097

[32] Tasserie J Uhrig L Sitt JD Manasova D Dupont M Dehaene S . Deep brain stimulation of the thalamus restores signatures of consciousness in a nonhuman primate model. Sci Adv. (2022) 8:eabl5547. doi: 10.1126/sciadv.abl5547, 35302854 PMC8932660

[33] Schiff ND Giacino JT Kalmar K Victor JD Baker K Gerber M . Behavioural improvements with thalamic stimulation after severe traumatic brain injury. Nature. (2007) 448:600–3. doi: 10.1038/nature06041, 17671503

[34] Schiff ND. Recovery of consciousness after brain injury: a mesocircuit hypothesis. Trends Neurosci. (2010) 33:1–9. doi: 10.1016/j.tins.2009.11.002, 19954851 PMC2931585

[35] Rolls ET Huang CC Lin CP Feng J Joliot M. Automated anatomical labelling atlas 3. NeuroImage. (2020) 206:116189. doi: 10.1016/j.neuroimage.2019.116189, 31521825

[36] Fonov V Evans AC Botteron K Almli CR McKinstry RC Collins DL . Unbiased average age-appropriate atlases for pediatric studies. NeuroImage. (2011) 54:313–27. doi: 10.1016/j.neuroimage.2010.07.033, 20656036 PMC2962759

[37] Rorden C Bonilha L Fridriksson J Bender B Karnath HO. Age-specific CT and MRI templates for spatial normalization. NeuroImage. (2012) 61:957–65. doi: 10.1016/j.neuroimage.2012.03.020, 22440645 PMC3376197

[38] Jeong H Song IU Chung YA Park JS Na SH Im JJ . Short-term efficacy of transcranial focused ultrasound to the Hippocampus in Alzheimer's disease: a preliminary study. J Pers Med. (2022) 12:250. doi: 10.3390/jpm12020250, 35207738 PMC8878180

[39] Legon W Ai L Bansal P Mueller JK. Neuromodulation with single-element transcranial focused ultrasound in human thalamus. Hum Brain Mapp. (2018) 39:1995–2006. doi: 10.1002/hbm.23981, 29380485 PMC6866487

[40] Delorme A Makeig S. EEGLAB: an open source toolbox for analysis of single-trial EEG dynamics including independent component analysis. J Neurosci Methods. (2004) 134:9–21. doi: 10.1016/j.jneumeth.2003.10.009, 15102499

[41] Penny WD Friston KJ Ashburner JT Kiebel SJ Nichols TE. Statistical parametric mapping: the analysis of functional Brain Images. Amsterdam: Elsevier. (2007).

[42] Giller CA Mornet P Moreau JF. The first formulation of image-based stereotactic principles: the forgotten work of Gaston Contremoulins. J Neurosurg. (2017) 127:1426–35. doi: 10.3171/2016.10.JNS161966, 28298020

[43] Tsubokawa T Yamamoto T Katayama Y Hirayama T Maejima S Moriya T. Deep-brain stimulation in a persistent vegetative state: follow-up results and criteria for selection of candidates. Brain Inj. (1990) 4:315–27. doi: 10.3109/02699059009026185, 2252964

[44] Magrassi L Maggioni G Pistarini C Di Perri C Bastianello S Zippo AG . Results of a prospective study (CATS) on the effects of thalamic stimulation in minimally conscious and vegetative state patients. J Neurosurg. (2016) 125:972–81. doi: 10.3171/2015.7.JNS15700, 26745476

[45] Schiff ND Giacino JT Butson CR Choi EY Baker JL O'Sullivan KP . Thalamic deep brain stimulation in traumatic brain injury: a phase 1, randomized feasibility study. Nat Med. (2023) 29:3162–74. doi: 10.1038/s41591-023-02638-4, 38049620 PMC11087147

[46] Demertzi A Antonopoulos G Heine L Voss HU Crone JS de Los Angeles C . Intrinsic functional connectivity differentiates minimally conscious from unresponsive patients. Brain. (2015) 138:2619–31. doi: 10.1093/brain/awv169, 26117367

[47] Di Perri C Bastianello S Bartsch AJ Pistarini C Maggioni G Magrassi L . Limbic hyperconnectivity in the vegetative state. Neurology. (2013) 81:1417–24. doi: 10.1212/WNL.0b013e3182a43b78, 24049132

[48] Stam CJ. Modern network science of neurological disorders. Nat Rev Neurosci. (2014) 15:683–95. doi: 10.1038/nrn3801, 25186238

[49] Arnts H Tewarie P van Erp W Schuurman R Boon LI Pennartz CMA . Deep brain stimulation of the central thalamus restores arousal and motivation in a zolpidem-responsive patient with akinetic mutism after severe brain injury. Sci Rep. (2024) 14:2950. doi: 10.1038/s41598-024-52267-1, 38316863 PMC10844373

[50] Darmani G Ramezanpour H Sarica C Annirood R Grippe T Nankoo JF . Individualized non-invasive deep brain stimulation of the basal ganglia using transcranial ultrasound stimulation. Nat Commun. (2025) 16:2693. doi: 10.1038/s41467-025-57883-7, 40108143 PMC11923056

