# Correction: The efficacy and safety of low-intensity focused ultrasound pulses for prolonged disorders of consciousness: a study protocol for a randomized controlled trial

**DOI:** 10.3389/fneur.2025.1751975

**Published:** 2026-01-06

**Authors:** Yuehong Huang, Qiuyi Yu, Hongxiang Liu, Chenxia Xue, Caroline Schnakers, Steven Laureys, Martin M. Monti, Haibo Di

**Affiliations:** 1 International Vegetative State and Consciousness Science Institute, Hangzhou Normal University, Hangzhou, China; 2 College of Life and Environmental Sciences, Hangzhou Normal University, Hangzhou, China; 3 Basical Department, Medical College, Hangzhou Normal University, Hangzhou, China; 4 Department of Intensive care rehabilitation, Shanghai Yangzhi Rehabilitation Hospital (Shanghai Sunshine Rehabilitation Center), Shanghai, China; 5 Research Institute, Casa Colina Hospital and Centers for Healthcare, Pomona, CA, United States; 6 Coma Science Group, GIGA Consciousness and Centre du Cerveau, University & University Hospital of Liège, Liège, Belgium; 7 Department of Psychology, University of California Los Angeles, Los Angeles, CA, United States; 8 Brain Injury Research Center (BIRC), Department of Neurosurgery, University of California Los Angeles, Los Angeles, CA, United States; 9 Department of Neurosurgery, University of California Los Angeles, Los Angeles, CA, United States

**Keywords:** low-intensity focused ultrasound, prolonged disorders of consciousness, randomized controlled trial, multi-modal measurement, rehabilitation

The order of authors in the author list of the published paper was erroneously given as:

“Haibo Di^1, 2*^, Yuehong Huang^1, 2, 3^, Qiuyi Yu^1, 2^, Hongxiang Liu^4^, Chenxia Xue^4^, Caroline Schnakers^5^, Steven Laureys^6^ and Martin M. Monti^7, 8, 9^”.

The correct order of the authors appears below:

“Yuehong Huang^1, 2, 3^, Qiuyi Yu^1, 3^, Hongxiang Liu^4^, Chenxia Xue^4^, Caroline Schnakers^5^, Steven Laureys^6^, Martin M. Monti^7, 8, 9^ and Haibo Di^1, 3*^”.

Authors Qiuyi Yu and Haibo Di was erroneously given as affiliation [1, 2]. The correct affiliation is [1, 3].

The original version of this article has been updated.

