# Supplementary material for: The efficacy and safety of low-intensity focused ultrasound pulses for prolonged disorders of consciousness: a study protocol for a randomized controlled trial
Source: Front Neurol. 2025 Nov 6;16:1597567. doi: 10.3389/fneur.2025.1597567 (PMC12631113; doi:10.3389/fneur.2025.1597567)
Supplement: Supplementary file 1 [file Data_Sheet_1.pdf]

Supplementary material 1

fMRI

The order of scanning is as follows(*T1+fMRI+DTI)*:the localization image will first be scanned. Next, task state and resting state functional images will be acquired using a gradient echo-planar imaging (EPI) sequence with parameters: scanning along the transverse plane; repetition time (TR) = 2000 ms; echo time (TE) = 30 ms; flip angle = 90° ; slices = 48; field of view (FOV) = 240 × 240mm^2; matrix size = 80 × 80; voxel size = 3.0 × 3.0 × 3.5 mm^3; and layer spacing of 20% of the layer thickness; call names task time = 4' 46''; resting state time = 8' 10''. Then diffusion tensor imaging (DTI) will be scanned, using a single excitation EPI sequence, with parameters: scanning along the transverse plane; TR =5600ms; TE = 88ms; flip angle = 90° ; slices = 46; FOV = 230 × 230 mm^2; matrix size = 128 × 128; voxel size = 1.8 × 1.8 × 3.0 mm^3; number of directions = 32; b-value = 1000 s/mm^2; b = 0 for 2 images; total time = 9' 58''. Finally, the T1-weighted images will be acquired by a multiplanar reconstruction sequence (magnetization-prepared, rapid acquisition gradient echo, MP-RAGE) with the following parameters: scanning along the sagittal plane; TR= 1800ms; TE= 4.21ms; flip angle = 9° ; slices = 176; FOV = 256 × 232mm^2; matrix size = 256 × 230; voxel size = 1.0 × 1.0 × 1.0 mm^3; time = 3' 59''. *Paradigm introduction*:*Call names:* We digitally record and adapt the subject's own name by a familiar voice (SON-FV) using the voice of a first-degree relative and GoldWave software. fMRI scanning will be performed using block design with nine active blocks and ten baseline blocks for each patient. Each active block lasted 12 seconds and included six SON-FVs (each name lasted 2 sec).

1H-MRS

Following the aforementioned T1-weighted sequence (with parameters as stated above), multivoxels which include several 30 × 30 × 30 mm^3 volume of interest (VOI) are placed in the left thalamus region (consistent with the target for LIFUP). The MEGA-PRESS sequence is utilized for measuring Glx/GABA concentration, with parameters as follows: TR=2000ms, TE=68ms, AV=96, BW=2000Hz, modified to BW=62.10Hz. By applying a Gaussian inversion pulse at the GABA CH2 resonance peak at 1.9 ppm (ON) and at the symmetric position of the water peak at 7.5 ppm (OFF), J-evolution focusing on GABA is achieved during odd-numbered acquisitions (ON), but no focusing is applied during even-numbered acquisitions (OFF). After automatic optimization, water suppression is performed using CHESS pulses. FASTMAP correction of the VOI is automatically conducted before each acquisition. The difference between the "ON" and "OFF" spectra provides the edited GABA spectrum, and MRS itself can measure the concentration of glutamate and glutamine(Glx) and other chemicals within the target region.

Brain PET-CT

The scanning parameters are as follows: axial PET (matrix = 128 × 128; pixel size = 1.95 × 1.95 mm^2; slice thickness = 3.27 mm) and CT images (FOV = 250 × 250 mm^2; matrix = 512 × 512; pixel size = 0.49 × 0.49 mm^2; slice thickness = 0.50 mm). Metlab is used for preprocessing and analysis of PET images. The presence of large calcifications or metal objects (>3 mm, half of the ultrasonic wavelength) within the cranial cavity is excluded, as these may interfere with the propagation of ultrasound waves.

SSEPs

The EMG machine (Keypoint9033A07, Natus Dantec, Denmark) is used to record the relevant waveforms. During the intervention treatment process, there is no stimulation protocol. During

periods when patients are not undergoing interventions, the stimulation sites are the median nerves at the wrists of the right upper limbs (located 2 cm proximal to the wrist crease at the midline), both prior to and following the intervention. The stimulation wave is a constant current monophasic pulse, with a stimulation intensity of 15–25 mA. The recording sites are the Erbs points on both upper limbs at the clavicle level, with neck electrodes placed at the levels of cervical vertebrae 6, scalp electrode recording points at C3′ and C4′, Fz as the reference electrode, and the back of the hand as the grounding line.

EEG

The specific method involves using a 64-channel EEG device(Biosemi,Active Two,Netherlands) for signal acquisition, with electrodes made of silver/silver chloride mixture. During data collection, the impedance between the skin and electrodes is maintained below 5 kΩ. Participants will be required to remain behaviorally awake throughout the data collection process. We will acquire resting-state EEG for 10 minutes and task-related EEG for the duration specified by the paradigm. The paradigm employed in this study is a modified version of the Local-Global paradigm[1]. As in the original paradigm, each trial consists of five consecutive tones with a stimulus onset asynchrony (SOA) of 150 ms. Each tone is a 50-ms sound composed of three sinusoidal frequencies—either Sound A (350, 700, and 1400 Hz) or Sound B (500, 1000, and 2000 Hz). The first four tones in each trial are always identical. Within a given block, the fifth tone is identical to the first four in 70% of the trials, and differs in the remaining 30%. Only blocks following the "XXXXX" rule are retained, as compared to the original Local-Global paradigm. An additional control block is incorporated into the modified design. In this block, the fifth tone differs from the first four in all trials. Half of the trials end with Sound B (e.g., AAAAB), and the other half end with Sound A (e.g., BBBBA). These two trial types are randomly intermixed within the control block. Each participant completes eight blocks in total, two of which are control blocks. Among the remaining six blocks, three blocks follow AAAAA rule and the other blocks follow BBBBB rule. The number of trials per block varies from 60 to 80. The entire experiment takes approximately 30 minutes to complete.

Supplementary material 2

k-Plan Planning Report

Plan Summary

Subject Information	
Name:	XXX
ID1 / ID2:	XXX
Date of Birth:	XXXXXXXX
Age:	57
Gender:	Male (including trans man)
Comments:	

Plan Information	
Plan Label:	groupX
Plan Comments:	
Transducer Model:	DPX-500-4-NOMINAL-V1
Transducer ID:	NEUROFUS-DPX-500-4-NOMINAL-V1
Transducer Manufacturer:	NeuroFUS
Transducer Type:	Annular Array
Number of Sonications:	1

Label	Frequency	$P_{tp,\ target}$	$I_{pa,\ target}$*	$P_{sptp,\ water}$	$I_{sppa,\ water}$*	$P_{sptp,\ in\ situ}$	$I_{sppa,\ in\ situ}$*	MI_{tc}
lifupA 1	500 kHz	294 kPa	2.88 W/cm^2	657 kPa	14.4 W/cm^2	314 kPa	3.29 W/cm^2	0.445

Label	Target ΔT	Max ΔT	Target TD	Max TD
lifupA 1	0.117 ℃	0.731 ℃	<0.1 CEM	<0.1 CEM

Sonication 1: lifupA

System Parameters	
Driving Frequency:	500 kHz
Focal Distance:	80 mm
Output Level Setting ($I_{sppa,\ water}$*):	14.4 W/cm^2
Cooling Time:	0 s

	Duration	Repetition Interval	Ramp Shape	Ramp Duration	$I_{spta,\ water}$*	$I_{spta,\ in\ situ}$*
Pulse	500 us	10 ms	No Ramp	0 s	720 mW/cm^2	165 mW/cm^2
Pulse Train	30 s	30 s	No Ramp	0 s	720 mW/cm^2	165 mW/cm^2
Repeat 1	600 s	600 s	No Ramp	0 s	720 mW/cm^2	165 mW/cm^2

Regional Peak Values			
	P_{sptp}	Max ΔT	Max Thermal Dose
background	250 kPa	209 m℃	<0.1 CEM
head	314 kPa	604 m℃	<0.1 CEM
skull	189 kPa	731 m℃	<0.1 CEM

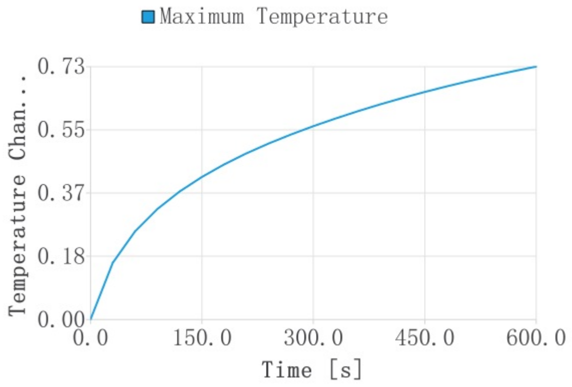

k-Plan Settings

k-Plan Information	
Plan Created Using:	k-Plan TPM v1.2.0
Plan Executed Using:	k-Plan SEM v1.2.1
Plan Executed On:	23 March 2025
Report Generated Using:	k-Plan TPM v1.2.0

Report Generated On:	3 April 2025
Report Generated By:	Peng Zhao

Simulation Settings	
Domain Size:	278 mm × 315 mm × 269 mm
Grid Spacing:	513 um
Grid Points Per Wavelength:	6
Output Downsampling Factor:	1
Grid Traversals:	2
Run Thermal Simulations:	Yes
Linked Sonications:	No
Material Property Conversion Method:	Head CT

Supplementary material 3

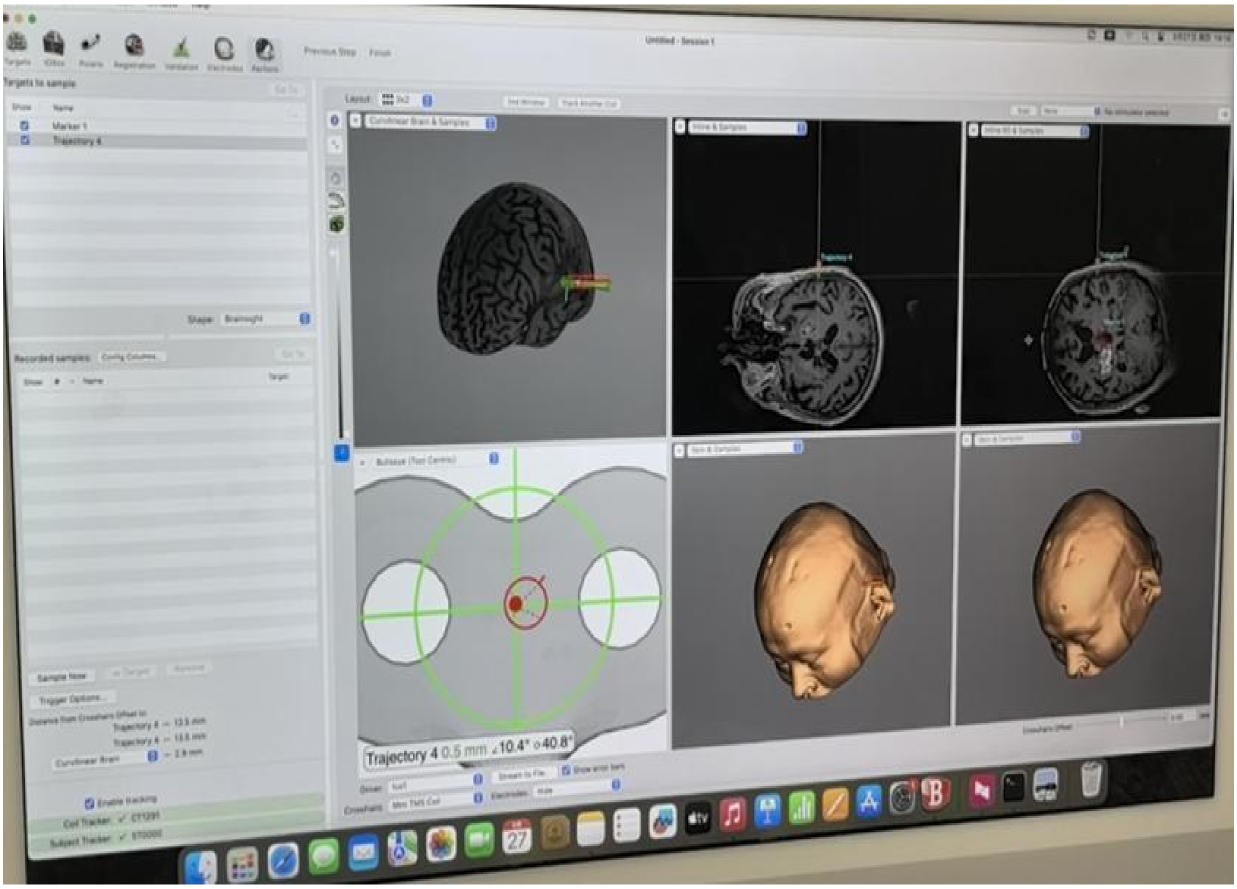

Supplementary material 4

Results

A 57-year-old man experienced a spontaneous hemorrhagic stroke at home and had remained in a state of disordered consciousness for 64 days since the time of injury (January 12th, 2025). His past medical history was notable for type 2 diabetes mellitus, coronary heart disease, and hypertension. Structural magnetic resonance imaging revealed a hyperintense lesion in the right thalamus and basal ganglia following the hemorrhage (Figure 7). At baseline, the patient exhibited behaviors consistent with a minimally conscious state minus (MCS-), including visual tracking in the upward, downward, and rightward directions. His CRS-R score was 9/23 (Table 1). Following the target localization protocol, we first localized the patient's left thalamus using the predefined target steps, as demonstrated by the color-highlighted region in Figure 7. Subsequently, we performed precise focal distance measurements via the simulator to generate simulation-based stimulation reports at varying distances. Based on these results, the optimal focal distance and transducer positioning were selected. Finally, the therapeutic intervention was administered according to the simulation-derived distance. We performed a simulated stimulation using the K-Plan system (Figure 8), with results detailed in Supplementary Material 02. Subsequently, two LIFUP stimulation sessions were administered using 100 Hz TUS (Group A), following the protocol detailed in the Procedure section.(see Supplementary Material 03 for photos taken during the intervention). Over the course of 20 days following treatment of LIFUP, the CRS-R score of the patient improved from 9 to 12/23 (Table 1), indicating a transition to a minimally conscious state plus (MCS+). New behavioral signs included reproducible responses to commands, as well as additional functions such as pain localization and auditory localization.

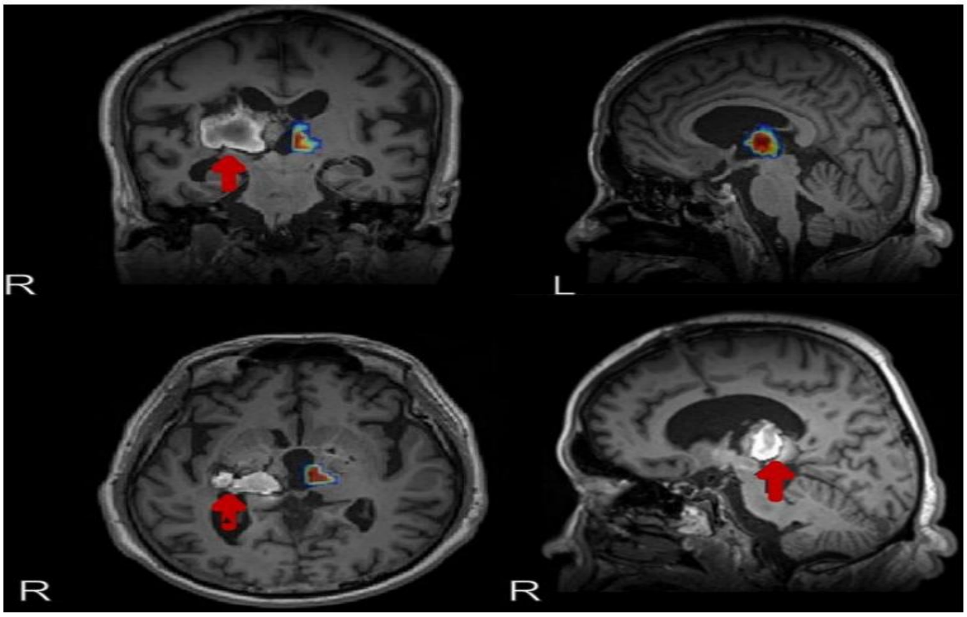

Figure.7. The red arrows represent the lesion sites of the right thalamus and basal ganglia.The highlighted colored areas on the left represent the target.

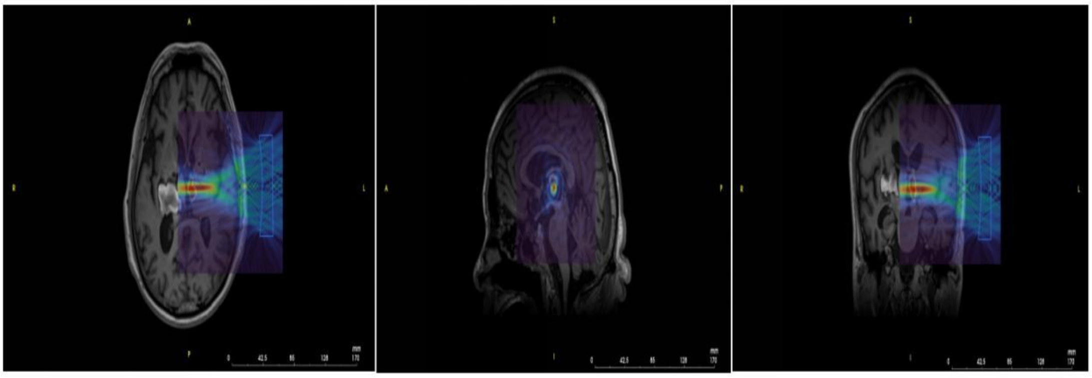

Figure.8. Pressure maps derived from ultrasound modeling superimposed on the MRI image of patient, highlighting the targeting of the left medial thalamus.

References：

1.Bekinschtein TA, Dehaene S, Rohaut B, Tadel F, Cohen L, Naccache L. Neural signature of the conscious processing of auditory regularities. Proc Natl Acad Sci U S A. 2009 Feb 3;106(5):1672-7. doi: 10.1073/pnas.0809667106. Epub 2009 Jan 21.
