# Supplementary material for: The efficacy and safety of low-intensity focused ultrasound pulses for prolonged disorders of consciousness: a study protocol for a randomized controlled trial
Source: Front Neurol. 2025 Nov 6;16:1597567. doi: 10.3389/fneur.2025.1597567 (PMC12631113; doi:10.3389/fneur.2025.1597567)
Supplement: Supplementary file 2 [file Table_1.docx]

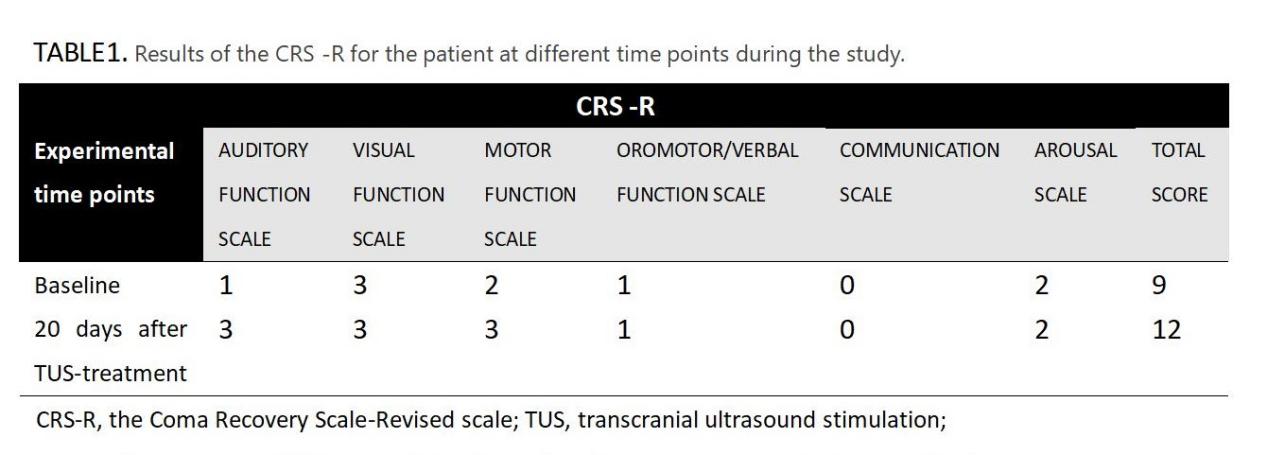


| **Experimental time points** | **CRS-R** | | | | | | |
| --- | --- | --- | --- | --- | --- | --- | --- |
|  | AUDITORY FUNCTION SCALE | VISUAL FUNCTION SCALE | MOTOR FUNCTION SCALE | OROMOTOR/VERBAL FUNCTION SCALE | COMMUNICATION SCALE | AROUSAL SCALE | TOTAL SCORE |
| Baseline | 1 | 3 | 2 | 1 | 0 | 2 | 9 |
| Before the first TUS | 1 | 3 | 2 | 1 | 0 | 2 | 9 |
| After the first TUS | 2 | 3 | 2 | 1 | 0 | 2 | 10 |
| Before the second TUS | 1 | 3 | 1 | 1 | 0 | 2 | 8 |
| After the second TUS | 3 | 3 | 3 | 1 | 0 | 2 | 12 |
